# Validation of the Japanese version of the Pediatric Quality of Life Inventory (PedsQL) Cancer Module

**DOI:** 10.1186/1477-7525-9-22

**Published:** 2011-04-10

**Authors:** Naoko Tsuji, Naoko Kakee, Yasushi Ishida, Keiko Asami, Ken Tabuchi, Hisaya Nakadate, Tsuyako Iwai, Miho Maeda, Jun Okamura, Takuro Kazama, Yoko Terao, Wataru Ohyama, Yuki Yuza, Takashi Kaneko, Atsushi Manabe, Kyoko Kobayashi, Kiyoko Kamibeppu, Eisuke Matsushima

**Affiliations:** 1 Section of Liaison Psychiatry and Palliative Medicine, Graduate School of Tokyo Medical and Dental University, 1-5-45 Yushima, Bunkyo-ku, Tokyo 113-8519, Japan; 2 Department of Hematology-Oncology, Tokyo Metropolitan Children's Medical Center, 2-8-29, Musashidai, Fuchu City, Tokyo 183-8561, Japan; 3 Department of Health Policy, National Research Institute for Child Health & Development, 10-1, Okura 2-chome, Setagaya, Tokyo 157-8535, Japan; 4 Department of Pediatrics, St. Luke's International Hospital, 9-1 Akashi-cho, Chuo-ku, Tokyo 104-8560, Japan; 5 Department of Pediatrics, Niigata Cancer Center Hospital, 2-15-3, Kawagishi-cho, Chuo-ku, Niigata City, Niigata 951-8566, Japan; 6 Department of Pediatrics, Kanagawa Children's Medical Center, 2-138-4, Mutsukawa, Minami-ku, Yokohama 232-8555, Japan; 7 Department of Pediatrics, Kanagawa Welfare Federation of Japan Agricultural Cooperatives, Sagamihara Kyodo Hospital, 2-8-18 Hashimoto, Midori-ku, Sagamihara, Kanagawa 252-5188, Japan; 8 Department of Hematology-Oncology, Kagawa Children's Hospital, 2603 Zentsuji-cho, Zentsuji City, Kagawa 765-8501, Japan; 9 Department of Pediatrics, Nippon Medical School, 1-1-5, Sendagi, Bunkyo-ku, Tokyo 113-8602, Japan; 10 Institute for Clinical Research, National Kyushu Cancer Center, 3-1-1, Notame, Minami-ku, Fukuoka 811-1395, Japan; 11 Department of Pediatric Surgery, Tohoku University, 28 Kawauchi, Aoba-ku, Sendai 980-8576, Japan; 12 Department of Family Nursing, Graduate School of Health Sciences and Nursing, Faculty of Medicine, The University of Tokyo, 7-3-1 Hongo, Bunkyo-ku, Tokyo 113-0033, Japan

## Abstract

**Background:**

The PedsQL 3.0 Cancer Module is a widely used instrument to measure pediatric cancer specific health-related quality of life (HRQOL) for children aged 2 to 18 years. We developed the Japanese version of the PedsQL Cancer Module and investigated its reliability and validity among Japanese children and their parents.

**Methods:**

Participants were 212 children with cancer and 253 of their parents. Reliability was determined by internal consistency using Cronbach's coefficient alpha and test-retest reliability using intra-class correlation coefficient (ICC). Validity was assessed through factor validity, convergent and discriminant validity, concurrent validity, and clinical validity. Factor validity was examined by exploratory factor analysis. Convergent and discriminant validity were examined by multitrait scaling analysis. Concurrent validity was assessed using Spearman's correlation coefficients between the Cancer Module and Generic Core Scales, and the comparison of the scores of child self-reports with those of other self-rating depression scales for children. Clinical validity was assessed by comparing the on- and off- treatment scores using Kruskal-Wallis and Mann-Whitney U tests.

**Results:**

Cronbach's coefficient alpha was over 0.70 for the total scale and over 0.60 for each subscale by age except for the 'pain and hurt' subscale for children aged 5 to 7 years. For test-retest reliability, the ICC exceeded 0.70 for the total scale for each age. Exploratory factor analysis demonstrated sufficient factorial validity. Multitrait scaling analysis showed high success rates. Strong correlations were found between the reports by children and their parents, and the scores of the Cancer Module and the Generic Core Scales except for 'treatment anxiety' subscales for child reports. The Depression Self-Rating Scale for Children (DSRS-C) scores were significantly correlated with emotional domains and the total score of the cancer module. Children who had been off treatment over 12 months demonstrated significantly higher scores than those on treatment.

**Conclusions:**

The results demonstrate the reliability and validity of the Japanese version of the PedsQL Cancer Module among Japanese children.

## Background

In the last 50 years, long-term survival rates of children with cancer have dramatically improved and 70 to 80% of patients can now be cured in developed countries [1]. However, 20 to 30% of patients who are diagnosed with advanced-stage neuroblastoma, soft tissue sarcoma, brainstem tumors, or relapsed tumors do not survive. For this reason, pediatric oncologists have 2 missions. For curable disease, we need to optimize anti-cancer treatment by reducing toxicity and preventing late complications without reducing the survival rate [2-6]. For fatal diseases, we have to balance the benefit and toxicity of anti-cancer treatment to maximize the quality of life remaining for the patients. To achieve both missions, we need to be able to measure the quality of life of childhood cancer patients. However, there has been no standardized measurement scale to do this in Japan.

The World Health Organization defined health as 'a state of complete physical, mental and social well-being and not merely the absence of disease or infirmity' [7]. Therefore, a health-related quality of life (HRQOL) instrument should include physical, mental, and social health dimensions [8,9]. Moreover, a pediatric HRQOL measurement needs to consider the cognitive development of the child and integrate child self-reports and parent proxy-reports [10]. Taking these points into account, the PedsQL [11] is thought to be suitable. This scale has been used in many countries to measure HRQOL in children and adolescents aged 2 to 18 years. Evaluation is conducted by both children and parents; children aged 5 to 18 years are asked to evaluate their own HRQOL (child self-report) and the parents of children aged 2 to 18 years are asked to evaluate their child's HRQOL (parent proxy-report). The PedsQL was designed using a modular approach to integrate the advantages of generic and disease-specific approaches [12,13]. Generic core scales enable the comparison of HRQOL of healthy children with those of ill children. In Japan, Kobayashi and her colleagues have developed the Japanese version of the PedsQL 4.0 Generic Core Scales [14]. We could have used this scale to assess HRQOL for children with cancer, but the instrument was not developed specifically for oncology patients. To enhance the measurement sensitivity for these patients, a cancer-specific module is necessary.

The PedsQL 3.0 Cancer Module was designed to measure HRQOL dimensions optimally for children with cancer. This instrument has already been validated in English [6], German [15], Portuguese [16], and Chinese [17]. However, until now, validation of the Japanese version has not been conducted.

The aim of this study was to demonstrate the reliability, validity, and feasibility of the Japanese version of the PedsQL 3.0 Cancer Module and compare scores by treatment status. As a result, Japanese children will be able to join international clinical trials and contribute to improvement of HRQOL of childhood cancer patients.

## Methods

### Scale development

Before starting this validation study, we obtained permission from Dr. James W. Varni (JWV) to translate the PedsQL 3.0 Cancer Module into Japanese using a standardized validation procedure [18]. Two Japanese translators competent in English independently translated PedsQL into Japanese. After discussion among translators and the authors, these forward translations were unified into a single version that was a conceptually equivalent translation of the original English version. Then, a professional bilingual translator (Japanese and English) performed backward translation of the first version from Japanese to English. Comparing the back-translated and original versions, minor changes were made to the first version. Then, we conducted pilot testing by using this modified version.

This Japanese version was tested on children and their parents (a total of 16 children and 20 parents). Then the researchers (NT or NK) looked at the responses on each questionnaire, checked how long it took to complete, and asked the subjects how well they understood the questions.

A final version of the Japanese version of the PedsQL Cancer Module was produced after modification of the pilot version. All translation procedures were reported to JWV, who reviewed the equivalence between the final Japanese version and the original English version.

### Study population

This validation study was developed in Japan from September 2006 through June 2010. We recruited children with cancer and their parents from 9 hospitals in Japan. Children were excluded from this study if they had comorbid disease or major developmental disorders. Families who did not agree to join this study were also excluded. Children aged 5 to 18 years who were diagnosed with cancer were included in this study, and the parents were included if their child was 2 to 18 years old.

### Procedure and measurements

The PedsQL 3.0 Cancer Module instrument includes 27 items with 8 subscales: pain and hurt (2 items), nausea (5 items), procedural anxiety (3 items), treatment anxiety (3 items), worry (3 items), cognitive problems (5 items), perceived physical appearance (3 items), and communication (3 items). The child instrument differs by age group: 5 to 7, 8 to 12, and 13 to 18 years. The parent's version also differs by child's age group: 2 to 4, 5 to 7, 8 to 12, and 13 to 18 years. The participants evaluated how often a particular problem occurred in the past month, using a 3-point Likert scale (0 = never, 2 = sometimes, 4 = often) for children 5 to 7 years and a 5-point Likert scale (0 = never, 1 = almost never, 2 = sometimes, 3 = often, 4 = almost always) for children 8 to 18 years and for the parents of all ages. For children aged 5 to 7 years, a Face Scale with 3 pictures varying from a smiling face to a sad face was used.

The PedsQL 4.0 Generic Core Scales includes 23 items with 4 subscales: physical functioning (8 items), emotional functioning (5 items), social functioning (5 items), and school functioning (5 items). The instrument for children differs by age group: 5 to 7, 8 to 12, and 13 to 18 years. The parent's version also differs by child's age group: 2 to 4, 5 to 7, 8 to 12, and 13 to 18 years. Similar to the PedsQL Cancer Module, a 3-point Likert scale is used for children 5 to 7 years old and a 5-point Likert scale is used for children 8 to 18 years old and for parents of children of all ages.

The questionnaire was self-administered for parents and children aged 8 to 18 years, and interviewer-administered for children aged 5 to 7 years. According to the original English version, the interviewer was the child's parent. After the parent completed the parent proxy report separately from their child, they read out the questions for the child's self-report and marked the answers. Parents and children aged 8 to 18 years completed the questionnaire independently after reading the instructions on their own. Parents were also questioned about their age, job, academic background, and economic status.

The child's physician answered questions about the patient's sex, date of birth, age, tumor pathology, date of diagnosis, date of completion of therapy (chemotherapy, radiation therapy, and surgery), existing comorbid disease or major developmental disorders, and whether the cancer was newly diagnosed or recurrent disease.

Participants were 282 families of children with cancer aged 2 to 18 years. Children aged 5 to 18 years answered the PedsQL child self-reports (n = 212) and the parents of children aged 2 to 18 years answered the PedsQL parent proxy-reports (n = 253). Eight children and their parents were excluded from the study because 1 patient was 20 years old, 6 patients were diagnosed with brain tumor, and 1 patient had Down syndrome. Finally, the questionnaires from 204 children and 245 parents were collected and analyzed.

Test-retest reliability was assessed at Tokyo Metropolitan Kiyose Children's Hospital (the predecessor of Tokyo Metropolitan Children's Medical Center). Forty families with children in stable condition according to their attending physician agreed to take a retest after 1 week. Finally, 28 children and 39 parents completed the questionnaires.

### Statistical analyses

Statistical analyses of the study were conducted by SPSS 16.0J for Windows (SPSS, Inc., Chicago, USA) and the significance level was set at 0.05. We used pair-wise case deletion for missing values, and if more than 50% of the items were missing, the score was not computed. Items were reverse-scored and linearly transformed to a 0 to 100 scale (0 = 100, 1 = 75, 2 = 50, 3 = 25, 4 = 0). Higher scores indicated better quality of life.

For characterization of the sample, Fisher's exact test was used to examine the differences by treatment status. Multiple regression analysis was done for the significant factors by Fisher's exact test. For descriptive analyses, we calculated the mean, standard deviation, median, minimum, and maximum scores and skewness.

Reliability was determined by internal consistency using Cronbach's coefficient alpha and test-retest reliability using Spearman's intra-class correlation coefficient (ICC). Internal consistency was considered good when Cronbach's coefficient alpha exceeded 0.70. ICC between the initial test and retest was measured according to the following values: 0.40 representing moderate, 0.60 good, and 0.80 excellent correlation.

Validity was assessed through factor validity, convergent and discriminant validity, concurrent validity, and clinical validity. Factor validity was examined by exploratory factor analysis. The extraction method was principle factor analysis. Rotation method was Promax with Kaiser normalization on the 27 items. Factor loading greater than 0.30 was regarded as significant.

Convergent and discriminant validity were examined by multitrait scaling analysis [19]. We calculated the range of correlation coefficients and the success rate of each scale. Concurrent validity was assessed by Spearman's correlation coefficient between the PedsQL 3.0 Cancer Module and the PedsQL 4.0 Generic Core Scales, and the comparison of the scores of child self-reports with those of other self-rating depression scales for children. We analyzed the correlations by Spearman rather than Pearson correlations because of non-normal distributions.

Initially, we predicted that the 'pain and hurt' and 'nausea' subscales of the Cancer Module were correlated with the physical health scale of the Generic Core Scales. Similarly, we predicted that the 'procedural anxiety,' 'treatment anxiety,' and 'worry' subscales of the Cancer Module were correlated with 'psychosocial health' and 'emotional functioning' subscales of the Generic Core Scales. 'Cognitive problems,' 'perceived physical appearance,' and 'communication' subscales of the Cancer Module were compared with the 'social functioning' and 'school functioning' subscales of the Generic Core Scales.

Moreover, we assessed the correlation of the 'procedural anxiety,' 'treatment anxiety,' and 'worry' subscales of the Cancer Module with the Depression Self-Rating Scale for Children (DSRSC) [20] and the Center for Epidemiologic Studies Depression scale (CES-D) [21]. These scales have already been translated into Japanese and the Japanese versions have been validated. DSRSC and CES-D scores of less than 15 were considered to be within the normal range and scores 16 or greater were suspicious for depression.

To assess clinical validity, we compared the total and subscale scores between on-treatment and off-treatment status by Kruskal-Wallis and Mann-Whitney U tests. Feasibility was determined by the amount of time required to complete the questionnaires and the percentage of missing values.

We calculated the sample size needed to produce medium correlation (0.30) in the examination of convergent and discriminant validity. We set the type I error at 1% and the statistical power at 90%; thus the calculated sample size was 154. We estimated that approximately 50 to 70% of participants would agree to participate, so we decided to administer this test to 220 to 308 parents and their children.

For the retest, sample size was calculated on the basis of an expected ICC from 0.60 to 0.80. Setting the type I error at 5% and the statistical power at 80%, calculated sample size was 13. We estimated that approximately 30 to 50% of retest questionnaires would be returned; thus we decided to administer the retest to 40 parents and their children.

### Ethical considerations

This study was approved by the Institutional Review Board (IRB) at each hospital. In our country, people are sensitive to direct expression about cancer, so we used alternate terms in introductory writings and questionnaires, such as the Japanese version of the Pediatric Quality of Life Inventory Brain Tumor Module [22]. For participation in this study, informed consent was required from all parents. For children aged 5 or over, informed assent was also required.

## Results

### Characterization of the sample

Participants' characteristics are shown in Table 1. The average age of the children was 10.5 years (Standard Deviation [SD] = 3.9 years) and 55.1% of the patients were male. One hundred sixty-six patients (76.8%) had hematological diseases, and the remaining patients (22.0%) had solid tumors. The guardians who answered the questionnaires were predominantly mothers (93.9%) and about half of them were 40 to 60 years old. On-treatment status means the patient was receiving medical treatment such as chemotherapy, radiation therapy, or surgery (n = 88; 35.9%). Off-treatment status means the patient completed all therapy by the time of the assessment (n = 155; 63.3%). In this study, half of the patients had been off treatment for over 12 months (n = 124; 50.6%). Even though medical fees were almost completely covered by public insurance in Japan, half of the guardians rated their economic level as 'low' because most mothers had to quit their job to take care of their children.

**Table 1:** Characterization of the sample

Subject	Child On-Tx (n = 88)		Child Off-Tx = <12 (n = 33)		Child Off Tx >12 (n = 124)		Total sample (n = 245)		
	
	n	%	n	%	n	%	n	%	*P *value
Age									0.002
2-4 (parents only)	23	26.1	6	18.2	12	9.7	41	16.7	
5-7	28	31.8	9	27.3	25	20.2	62	25.3	
8-12	16	18.2	12	36.4	47	37.9	75	30.6	
13-18	21	23.9	6	18.2	40	32.3	67	27.3	

Sex									0.357
Male	51	58.0	21	63.6	63	50.8	135	55.1	
Female	37	42.0	12	36.4	61	49.2	110	44.9	

Diagnosis									0.002
Newly diagnosed	67	76.1	27	81.8	115	92.7	209	85.3	
Recurrent disease	21	23.9	6	18.2	9	7.3	36	14.7	

Tumor pathology									0.050
Leukemia	70	79.5	21	63.6	75	60.5	166	67.8	
Malignant lymphoma	7	8.0	4	12.1	11	8.9	22	9.0	
Neuroblastoma	4	4.5	2	6.1	11	8.9	17	6.9	
Wilms tumor	3	3.4	0	0	8	6.5	11	4.5	
Rhabdomyosarcoma	0	0	1	3.0	3	9.7	4	1.6	
Hepatoblastoma	1	1.1	0	0	2	2.4	3	1.2	
Other solid tumors	2	2.3	3	9.1	14	11.3	19	7.8	
Unknown	1	1.1	2	6.1	0	0	3	1.2	

Relationship to patient									0.257
Mother	80	90.9	32	97.0	118	95.2	230	93.9	
Father	3	3.4	1	3.0	5	4.0	9	3.7	
Other guardian	0	0	0	0	0	0	0	0	
Unknown	5	5.7	0	0	1	0.8	6	2.4	

Age of guardian									0.030
21-28	1	1.1	0	0	4	3.2	5	2.0	
29-34	17	19.3	7	21.2	16	12.9	40	16.3	
35-39	32	36.4	12	36.4	28	22.6	72	29.4	
40-60	33	37.5	13	39.4	74	59.7	120	49.0	
Unknown	5	5.7	1	3.0	2	1.6	8	3.3	

Guardian's academic background									0.065
Junior high school	3	3.4	0	0	1	0.8	4	1.6	
High school	32	36.4	14	42.4	41	33.1	87	35.5	
Vocational school	13	14.8	2	6.1	29	23.4	44	18.0	
Junior college	20	22.7	6	18.2	22	17.7	48	19.6	
University	14	15.9	10	30.3	28	22.6	52	21.2	
Graduate school	0	0	1	3.0	0	0	1	0.4	
Other	1	1.1	0	0	1	0.8	2	0.8	
Unknown	5	5.7	0	0	2	1.6	7	2.9	

Guardian's evaluation of economic level									0.485
Very high	1	1.1	0	0	4	3.2	5	2.0	
High	23	26.1	13	39.4	35	28.2	71	29.0	
Low	44	50.0	16	48.5	65	52.4	125	51.0	
Very low	14	15.9	4	12.1	18	14.5	36	14.7	
Unknown	6	6.8	0	0	2	1.6	8	3.3	

On-Tx: on treatment sample; Off-Tx = < 12: off treatment = < 12 months sample; Off-Tx > 12: off treatment > 12 months sample. *P *value is calculated by Fisher's exact test.

There was no statistically significant difference in the ratio of patient's sex, guardians who answered the questionnaires, their academic background, or their evaluation of economic level by treatment status.

For significant factors such as children's age, diagnosis, and age of guardian, multiple regression analysis was done (Table 2). None of the comparisons were statistically significant for the total score of the PedsQL Cancer Module, so that we considered the 3 treatment groups to have the same patient characteristics.

**Table 2:** Multivariable analysis of the total score of the PedsQL Cancer Module

Factor	SE	β	t	*P *value
Age	.362	.051	.556	.579
2-4 (parents only)				
5-7				
8-12				
13-18				

Diagnosis	2.866	-.108	-1.529	.128
Newly diagnosed				
Recurrent disease				

Age of guardian	.242	.155	1.673	.096
21-28				
29-34				
35-39				
40-60				
Unknown				

Treatment status	1.198	.298	4.207	<.0001
Child On Tx (n = 88)				
Child Off Tx = < 12 (n = 33)				
Child Off Tx > 12 (n = 124)				

Calculations were done by multiple regression analysis.

SE: standard error of the mean.

On Tx: on treatment sample; Off Tx = < 12: off treatment = < 12 months sample; Off Tx > 12: off treatment >12 months sample.

### Descriptive analysis

The child self-reports and the parent proxy-reports showed comparatively good concordance in all scales (Tables 3 and 4). Scale scores were consistently higher for child reports than for parent reports. For both child and parent reports, 'pain and hurt,' 'nausea,' and 'treatment anxiety' had higher scores than other subscale scores for all ages. On the other hand, the subscale 'communication' had a tendency to be low for all ages. However, the scores for 'cognitive problems' and 'perceived physical appearance' were lowest in adolescents (13-18 y).

**Table 3:** Score distributions of the Japanese version of the PedsQL Cancer Module (Child self-report)

Subscale	n	mean	(SD, range)	α	floor	ceiling	skewness
Total	193	77.89	(15.35, 29.79-100)	0.78	62.54	93.24	-.620
Pain and hurt	202	84.72	(19.66, 0-100)	0.72	65.06	104.38	-1.177
Nausea	199	82.96	(23.96, 0-100)	0.88	59.00	106.92	-1.548
Procedural anxiety	203	72.90	(30.96, 0-100)	0.87	41.94	103.86	-1.032
Treatment anxiety	203	93.14	(17.01, 0-100)	0.84	76.13	110.15	-3.400
Worry	202	76.61	(25.91, 0-100)	0.80	50.70	102.52	-1.101
Cognitive problems	201	72.39	(22.09, 6.25-100)	0.72	50.30	94.48	-.546
Perceived physical appearance	204	70.34	(28.58, 0-100)	0.75	41.76	98.92	-.797
Communication	204	67.03	(27.01, 0-100)	0.74	40.02	94.04	-.596

2-4 years							
Total							
Pain and hurt							
Nausea							
Procedural anxiety							
Treatment anxiety				NA			
Worry							
Cognitive problems							
Perceived physical appearance							
Communication							

5-7 years							
Total	58	73.27	(14.57, 43.33-100)	0.67	58.70	87.84	.039
Pain and hurt	61	84.02	(19.38, 50-100)	0.53	64.64	103.40	-.735
Nausea	61	76.72	(23.86, 0-100)	0.82	52.86	100.58	-1.295
Procedural anxiety	62	55.11	(36.91, 0-100)	0.88	18.20	92.02	-.159
Treatment anxiety	61	88.25	(22.62, 0-100)	0.79	65.63	110.87	-2.275
Worry	60	73.61	(28.01, 0-100)	0.73	45.60	101.62	-.915
Cognitive problems	60	73.13	(23.11, 12.5-100)	0.67	50.02	96.24	-.572
Perceived physical appearance	62	70.43	(28.22, 0-100)	0.67	42.21	98.65	-.786
Communication	62	59.95	(26.90, 0-100)	0.60	33.05	86.85	-.422

8-12 years							
Total	72	79.36	(15.94, 32.71-100)	0.82	63.42	95.30	-.923
Pain and hurt	75	86.17	(20.51, 0-100)	0.84	65.66	106.68	-1.825
Nausea	73	83.84	(25.65, 5-100)	0.91	58.19	109.49	-1.715
Procedural anxiety	75	78.22	(27.57, 0-100)	0.89	50.65	105.79	-1.393
Treatment anxiety	75	94.56	(14.14, 25-100)	0.83	80.42	108.70	-3.636
Worry	75	78.78	(25.79, 0-100)	0.83	52.99	104.57	-1.130
Cognitive problems	74	71.35	(20.70, 5-100)	0.72	50.65	92.05	-.600
Perceived physical appearance	75	72.00	(29.69, 0-100)	0.80	42.31	101.69	-.906
Communication	75	66.67	(28.08, 0-100)	0.76	38.59	94.75	-.590

13-18 years							
Total	62	80.25	(14.79, 29.79-100)	0.82	65.46	95.04	-.925
Pain and hurt	66	83.71	(19.11, 37.5-100)	0.75	64.60	102.82	-.799
Nausea	65	87.85	(20.97, 10-100)	0.90	66.88	108.82	-1.775
Procedural anxiety	66	83.59	(19.61, 25-100)	0.69	63.98	103.20	-1.162
Treatment anxiety	67	96.02	(13.71, 0-100)	0.94	82.31	109.73	-5.666
Worry	67	76.87	(24.18, 0-100)	0.85	52.69	101.05	-1.330
Cognitive problems	66	70.30	(23.20, 20-100)	0.82	47.10	93.50	-.305
Perceived physical appearance	67	68.41	(27.96, 0-100)	0.81	40.45	96.37	-.735
Communication	67	74.01	(24.38, 0-100)	0.83	49.63	98.39	-.810

n: number of individuals, SD: standard deviation, α: Cronbach's coefficient.

**Table 4:** Score distributions of the Japanese version of the PedsQL Cancer Module (Parent proxy-report)

Subscale	n	mean	(SD, range)	α	floor	ceiling	skewness
Total	188	74.91	(15.25, 24.95-100)	0.79	59.66	90.16	-.573
Pain and hurt	242	82.85	(22.00, 0-100)	0.89	60.85	104.85	-1.221
Nausea	233	80.49	(25.70, 0-100)	0.93	54.79	106.19	-1.324
Procedural anxiety	242	63.19	(31.76, 0-100)	0.92	31.43	94.95	-.503
Treatment anxiety	241	84.89	(19.00, 0-100)	0.90	65.89	103.89	-1.352
Worry	242	81.37	(21.91, 0-100)	0.87	59.46	103.28	-1.321
Cognitive problems	203	68.78	(21.61, 8.33-100)	0.84	47.17	90.39	-.470
Perceived physical appearance	243	73.77	(24.92, 0-100)	0.86	48.85	98.69	-.903
Communication	241	62.21	(25.42, 0-100)	0.81	36.79	87.63	-.416

2-4 years							
Total	38	76.31	(16.37, 40.83-100)	0.81	59.94	92.68	-.478
Pain and hurt	41	86.89	(18.32, 25-100)	0.83	68.57	105.21	-1.365
Nausea	39	72.18	(24.78, 30-100)	0.91	47.40	96.96	-.140
Procedural anxiety	40	58.13	(35.03, 0-100)	0.89	23.10	93.16	-.213
Treatment anxiety	41	75.61	(26.51, 0-100)	0.94	49.10	102.12	-.849
Worry	41	87.60	(22.52, 0-100)	0.93	65.08	110.12	-2.110
Cognitive problems	40	78.13	(20.03, 25-100)	0.88	58.10	98.16	-.607
Perceived physical appearance	40	83.54	(23.76, 16.67-100)	0.91	59.78	107.30	-1.571
Communication	40	65.83	(28.48, 0-100)	0.78	37.35	94.31	-.701

5-7 years							
Total	56	73.70	(13.04, 39.32-100)	0.68	60.66	86.74	-.114
Pain and hurt	61	84.63	(19.15, 37.50-100)	0.79	65.48	103.78	-.893
Nausea	59	78.98	(27.34, 0-100)	0.94	51.64	106.32	-1.530
Procedural anxiety	62	47.58	(33.11, 0-100)	0.93	14.47	80.69	.102
Treatment anxiety	61	83.47	(17.58, 25-100)	0.85	65.89	101.05	-1.091
Worry	61	84.97	(17.80, 33.33-100)	0.80	67.17	102.77	-1.061
Cognitive problems	62	70.87	(19.89, 6.25-100)	0.87	50.98	90.76	-.402
Perceived physical appearance	62	76.61	(21.12, 0-100)	0.84	55.49	97.73	-1.018
Communication	61	58.20	(25.84, 0-100)	0.85	32.36	84.04	-.320

8-12 years							
Total	71	74.26	(16.48, 25.42-98.75)	0.82	57.78	90.74	-.855
Pain and hurt	75	81.00	(25.78, 0-100)	0.94	55.22	106.78	-1.376
Nausea	72	82.99	(26.48, 0-100)	0.95	56.51	109.47	-1.637
Procedural anxiety	75	68.56	(28.59, 0-100)	0.94	39.97	97.15	-.868
Treatment anxiety	74	87.16	(17.07, 33.33-100)	0.84	70.09	104.23	-1.443
Worry	75	79.00	(24.21, 0-100)	0.87	54.79	103.21	-1.309
Cognitive problems	75	64.80	(22.09, 5-100)	0.83	42.71	86.89	-.190
Perceived physical appearance	75	69.11	(25.99, 0-100)	0.82	43.12	95.10	-.745
Communication	74	60.92	(24.71, 0-100)	0.80	36.21	85.63	-.458

13-18 years							
Total	61	76.41	(15.57, 39.06-100)	0.84	60.84	91.98	-.416
Pain and hurt	65	80.77	(21.88, 25-100)	0.90	58.89	102.65	-.835
Nausea	63	84.21	(22.95, 5-100)	0.93	61.26	107.16	-1.631
Procedural anxiety	65	75.00	(25.17, 0-100)	0.88	49.83	100.17	-.709
Treatment anxiety	65	89.49	(14.45, 50-100)	0.92	75.04	103.94	-1.046
Worry	65	76.79	(21.22, 0-100)	0.86	55.57	98.01	-1.016
Cognitive problems	66	67.95	(23.60, 15-100)	0.89	44.35	91.55	-.445
Perceived physical appearance	66	70.45	(26.16, 0-100)	0.86	44.29	96.61	-.741
Communication	66	65.15	(23.75, 0-100)	0.85	41.40	88.90	-.271

n: number of individuals, SD: standard deviation, α: Cronbach's coefficient.

### Reliability

Cronbach's coefficient alpha for the total scale and each subscale exceeded 0.70 in both the child self-reports and parent proxy-reports (Tables 3 and 4). However, for children aged 5 to 7 years, Cronbach's coefficient alpha ranged from 0.53 to 0.67 in the 'pain and hurt,' 'cognitive problems,' 'perceived physical appearance,' and 'communication' subscales in self-reports.

Table 5 shows test-retest reliability analysis of the PedsQL Cancer Module scales in each age group. ICC values among the children ranged from good to excellent except for the 'treatment anxiety' subscale for 5- to 7-year-olds and 13- to 18-year-olds and the 'worry' subscale for 8- to 12-year-olds. ICC values among the parents ranged from good to excellent.

**Table 5:** Test-retest reliability of the Japanese version of the PedsQL Cancer Module

	2-4 years α ICC	5-7 years α ICC	8-12 years α ICC	13-18 years α ICC
Child self-report (n = 19)				
Pain and hurt		.42 .54	.38 .94**	.94 .94**
Nausea		.49 .80**	.86 .50	.92 .99**
Procedural anxiety		.72 .97**	.86 .46	.64 .67
Treatment anxiety	NA	-.06 -.12	.94 .76*	.91 .20
Worry		.90 .85**	.94 .20	.74 .92**
Cognitive problems		.66 .79**	.75 .74	.84 .93**
Perceived physical appearance		.79 .87**	.75 .45	.90 .97**
Communication		.83 .76**	.81 .85*	.92 .78*
Total		.79 .83**	.68 .79*	.85 1.00**

Parent proxy report (n = 38)				
Pain and hurt	.92 .86**	.85 .72**	.95 .99**	.99 .99**
Nausea	.95 .92**	.95 .83**	.89 1.00**	.98 .92*
Procedural anxiety	.98 .97**	.98 .95**	.96 .87*	.84 .75
Treatment anxiety	.81 .68*	.42 .34	.85 .74	.95 .89**
Worry	.95 .94**	.72 .51	.97 .87*	.95 .87**
Cognitive problems	.94 .90**	.92 .73**	.83 .71	.89 .92**
Perceived physical appearance	.94 .92**	.88 .86**	.82 .65	.94 .79*
Communication	.89 .81**	.88 .80**	.25 .25	.73 .71*
Total	.98 .97**	.92 .71*	.89 .86*	.93 1.00**

α: Cronbach's coefficient alpha, ICC: intraclass correlation coefficient, NA: not applicable, *P = < 0.05, **P = < 0.01 (2-tailed)

### Validity

Validity was assessed through factor validity, convergent and discriminant validity, concurrent validity, and clinical validity. Although the original English version has an 8-factor structure [11], exploratory factor analysis identified 7 factors for both child self-report and parent proxy-report in our Japanese version (Tables 6 and 7). The first item of 'worry' (worrying about side effects from medical treatments) loaded on the 'nausea' factor, and the second and third items of 'worry' (worrying about whether the medical treatments were working and worrying about reoccurrence or relapse) loaded on the 'communication' factor in the child self-report. Moreover, the first item of 'cognitive problems' (difficulty figuring out what to do when something bothers him/her) loaded on the 'perceived physical appearance' factor. In the parent-proxy report, the first and the second items of 'worry' loaded on the 'nausea' factor, and the third item loaded on the 'treatment anxiety' and 'perceived physical appearance' factors. Factor-item correlations were between 0.30 and 1.00 in the child self-reports, and 0.44 and 1.00 in the parent proxy-reports.

**Table 6:** Exploratory factor analysis of the PedsQL Cancer Module in child self-reports

Subscale	Item	Factor 1	Factor 2	Factor 3	Factor 4	Factor 5	Factor 6	Factor 7
Pain and hurt	P1	-.08	.13	-.10	.07	-.06	-.06	**.94**
	P2	.07	-.07	.03	-.02	.06	.01	**.77**

Nausea	N1	**.85**	.02	-.03	.13	-.06	-.06	.03
	N2	**.89**	.04	.03	-.07	.05	-.07	-.03
	N3	**.59**	.20	-.06	-.06	.15	.02	-.11
	N4	**.85**	.00	.07	.16	-.17	.04	.05
	N5	**.98**	.01	-.09	.01	-.08	.01	-.08

Procedural anxiety	PA1	.17	.11	-.03	**.62**	.17	-.17	.04
	PA2	-.03	-.13	.09	**.87**	-.10	.11	.05
	PA3	.03	-.05	.00	**.83**	-.01	.12	-.02

Treatment anxiety	TA1	-.07	.04	**.87**	.10	.12	-.09	-.08
	TA2	-.02	-.02	**1.00**	-.08	-.10	.07	.01
	TA3	.06	.05	**.67**	.08	.10	-.05	-.03

Worry	W1	**.51**	-.10	.08	-.05	.29	.10	.12
	W2	.20	-.14	.14	-.11	**.64**	.03	.07
	W3	.21	-.20	.01	-.17	**.59**	.09	.05

Cognitive problems	CP1	-.07	.16	-.05	.01	.22	**.30**	.22
	CP2	-.04	**.54**	-.09	.01	.22	.05	-.08
	CP3	.12	**.73**	-.07	-.01	.04	-.17	.03
	CP4	-.02	**.54**	.11	-.03	-.01	.04	.14
	CP5	.05	**.70**	.18	-.12	-.14	.20	.01

Perceived physical appearance	A1	.19	.22	.00	-.10	.02	**.41**	.02
	A2	-.01	-.12	.02	.02	.05	**.82**	-.05
	A3	-.06	.12	-.05	.12	-.05	**.81**	-.02

Communication	C1	-.14	.23	-.02	-.02	**.75**	-.02	-.06
	C2	-.11	.20	.08	.19	**.67**	-.14	.00
	C3	-.02	.04	-.10	.18	**.48**	.30	-.12

Extraction method is principle factor analysis by Promax rotation with Kaiser normalization.

Factor loading greater than 0.30 shown in boldface.

**Table 7:** Exploratory factor analysis of the PedsQL Cancer Module in parent proxy-reports

Subscale	Item	Factor 1	Factor 2	Factor 3	Factor 4	Factor 5	Factor 6	Factor 7
Pain and hurt	P1	-.01	.04	.00	-.04	.17	-.04	**.85**
	P2	.11	-.03	.01	.08	-.06	.03	**.93**

Nausea	N1	**.87**	-.08	.03	.03	-.05	-.02	.11
	N2	**.94**	.03	.01	-.11	-.08	.03	.08
	N3	**.60**	-.02	.08	.17	.16	.03	-.13
	N4	**1.00**	-.01	.03	-.02	-.18	.06	.04
	N5	**1.00**	-.01	-.07	-.10	-.05	.01	-.04

Procedural anxiety	PA1	.10	.07	**.85**	-.08	-.04	-.03	.00
	PA2	-.13	-.02	**.90**	.15	.02	-.09	.01
	PA3	.06	.00	**.95**	-.08	.00	.07	.00

Treatment anxiety	TA1	-.05	-.06	.12	**.83**	.00	.04	.02
	TA2	.08	.13	-.11	**.85**	-.18	.09	-.02
	TA3	-.06	.02	.00	**.90**	-.14	.08	.06

Worry	W1	**.66**	.00	.07	.08	.16	.00	-.06
	W2	**.45**	.00	-.05	.27	.27	-.13	-.01
	W3	.13	-.15	-.01	**.44**	**.49**	-.24	-.06

Cognitive problems	CP1	.04	**.55**	.07	.09	.09	.11	.02
	CP2	-.03	**.75**	.05	-.12	.03	.03	-.04
	CP3	-.11	**.89**	-.02	.01	-.12	-.12	.11
	CP4	-.01	**.77**	-.06	.17	.00	-.06	.02
	CP5	.08	**.86**	.05	-.02	.01	.01	-.10

Perceived physical appearance	A1	.27	.24	-.10	-.08	**.50**	-.01	.07
	A2	-.13	-.10	.07	-.09	**.83**	.06	.12
	A3	.00	.04	-.06	-.18	**.95**	.08	-.04

Communication	C1	.11	.01	-.01	.03	.02	**.86**	-.04
	C2	-.04	-.09	-.04	.10	.09	**.93**	.03
	C3	-.17	.12	.07	.12	.38	.29	.02

Extraction method is principle factor analysis by Promax rotation with Kaiser normalization.

Factor loading greater than 0.30 shown in boldface.

Convergent and discriminant validity were examined by multitrait scaling analysis (Table 8). After excluding item duplication, we calculated correlation coefficients between each item and the subscale that it belonged to. The success rate was determined by the percentage of items where the convergent correlation exceeded the discriminant correlation. All scales demonstrated extremely high success rates ranging from 95 to 100% in all ages.

**Table 8:** Multitrait scaling analysis of the PedsQL Cancer Module

		Childen			Parents	
Subscale	Convergent validity	Discriminant validity	Success rate	Convergent validity	Discriminant validity	Success rate
Total	0.46-0.83	0.02-0.61	99.5%	0.51-0.92	0.03-0.62	100%
Pain and hurt	0.56	0.06-0.44	100%	0.80	0.06-0.47	100%
Nausea	0.56-0.80	0.14-0.48	100%	0.66-0.92	0.18-0.62	100%
Procedural anxiety	0.72-0.83	0.02-0.35	100%	0.80-0.89	0.03-0.51	100%
Treatment anxiety	0.69-0.75	0.08-0.39	100%	0.79-0.81	0.11-0.52	100%
Worry	0.62-0.67	0.12-0.61	100%	0.70-0.83	0.15-0.60	100%
Cognitive problems	0.46-0.67	0.04-0.47	98.0%	0.62-0.77	0.03-0.45	100%
Perceived physical appearance	0.48-0.68	0.09-0.42	100%	0.66-0.80	0.16-0.45	100%
Communication	0.46-0.68	0.14-0.44	100%	0.51-0.79	0.19-0.42	100%

2-4 years						
Total				0.28-0.94	0.01-0.81	99.0%
Pain and hurt				0.77	-0.01-0.60	100%
Nausea				0.57-0.94	0.14-0.62	98.0%
Procedural anxiety				0.64-0.90	-0.01-0.81	96.0%
Treatment anxiety		NA		0.84-0.86	0.01-0.80	100%
Worry				0.78-0.94	0.02-0.54	100%
Cognitive problems				0.70-0.91	0.13-0.55	100%
Perceived physical appearance				0.54-0.62	0.19-0.56	100%
Communication				0.28-0.72	0.01-0.61	96.0%

5-7 years						
Total	0.31-0.88	0.00-0.49	99.0%	0.57-0.91	0.00-0.56	100%
Pain and hurt	0.39	0.00-0.35	100%	0.59	0.00-0.38	100%
Nausea	0.50-0.71	0.00-0.38	100%	0.68-0.91	0.02-0.56	100%
Procedural anxiety	0.67-0.88	0.03-0.41	100%	0.78-0.88	-0.01-0.41	100%
Treatment anxiety	0.66-0.70	0.01-0.46	100%	0.71-0.77	0.08-0.45	100%
Worry	0.46-0.65	-0.02-0.43	100%	0.60-0.71	0.00-0.52	100%
Cognitive problems	0.39-0.54	0.01-0.49	97.0%	0.63-0.83	0.00-0.42	100%
Perceived physical appearance	0.31-0.56	0.03-0.44	96.0%	0.63-0.81	0.08-0.43	100%
Communication	0.31-0.54	0.02-0.40	96.0%	0.57-0.79	0.00-0.43	100%

8-12 years						
Total	0.47-0.97	0.00-0.90	100%	0.43-0.87	0.01-0.78	98.0%
Pain and hurt	0.66	0.12-0.54	100%	0.88	0.07-0.57	100%
Nausea	0.79-0.99	0.11-0.65	100%	0.77-0.93	0.05-0.78	98.0%
Procedural anxiety	0.97-0.98	0.11-0.90	100%	0.87-0.96	-0.02-0.35	100%
Treatment anxiety	0.97-0.98	0.12-0.41	100%	0.69-0.73	0.13-0.52	100%
Worry	0.95-0.97	0.26-0.55	100%	0.64-0.88	-0.02-0.77	96.0%
Cognitive problems	0.94-0.98	0.00-0.44	100%	0.51-0.77	0.01-0.77	98.0%
Perceived physical appearance	0.93-0.96	0.11-0.45	100%	0.52-0.80	0.08-0.43	100%
Communication	0.47-0.65	0.15-0.46	100%	0.43-0.79	0.11-0.45	96.0%

13-18 years						
Total	0.51-0.91	0.08-0.64	98.0%	0.48-0.92	0.13-0.56	100%
Pain and hurt	0.71	0.15-0.46	100%	0.81	0.26-0.50	100%
Nausea	0.62-0.86	0.08-0.58	100%	0.48-0.92	0.16-0.55	98.0%
Procedural anxiety	0.51-0.75	0.10-0.42	100%	0.78-0.87	0.17-0.50	100%
Treatment anxiety	0.86-0.91	0.13-0.51	100%	0.81-0.91	0.24-0.56	100%
Worry	0.67-0.83	0.19-0.54	100%	0.71-0.87	0.22-0.53	100%
Cognitive problems	0.54-0.69	0.06-0.57	95.0%	0.65-0.81	0.14-0.53	100%
Perceived physical appearance	0.52-0.72	0.24-0.64	96.0%	0.73-0.76	0.25-0.48	100%
Communication	0.56-0.79	0.19-0.58	96.0%	0.59-0.81	0.13-0.51	100%

Convergent and discriminant validity is calculated by Pearson correlation coefficient, NA: not applicable

We calculated intraclass correlation coefficients between the child self-reports and parent proxy-reports (Table 9). For the entire sample, strong correlations ranging from 0.50 to 0.79 were demonstrated between the same subscales. Physical health scales ('pain and hurt' and 'nausea') demonstrated the strongest correlations.

**Table 9:** Intraclass Correlation Coefficients between child self-reports and parent proxy-reports in PedsQL Cancer Module

Children					Parents				
	P	N	PA	TA	W	CP	A	C	Total
Pain and hurt (P)	0.69**	0.35**	0.06	0.19**	0.35**	0.21**	0.25**	0.22**	0.44**
Nausea (N)	0.38**	0.79**	0.21**	0.27**	0.50**	0.21**	0.34**	0.30**	0.53**
Procedural anxiety (PA)	0.09	0.21**	0.73**	0.29**	0.03	0.04	0.10	0.17**	0.31**
Treatment anxiety (TA)	0.09	0.19**	0.26**	0.50**	0.21**	0.08	0.17**	0.21**	0.33**
Worry (W)	0.27**	0.48**	0.17**	0.28**	0.57**	0.23**	0.35**	0.37**	0.46**
Cognitive problems (CP)	0.18**	0.14*	0.04	0.12*	0.16*	0.60**	0.24**	0.30**	0.31**
Perceived physical appearance (A)	0.21**	0.24**	0.16*	0.25**	0.32**	0.25**	0.57**	0.29**	0.37**
Communication (C)	0.14*	0.31**	0.25**	0.29**	0.30**	0.36**	0.33**	0.60**	0.44**
Total	0.35**	0.47**	0.32**	0.43**	0.43**	0.35**	0.41**	0.44**	0.68**

*P = < 0.05, **P = < 0.01 (2-tailed)

Concurrent validity was assessed 2 ways. First, we compared Spearman's correlation coefficients between the PedsQL 3.0 Cancer Module and the PedsQL 4.0 Generic Core Scales (Table 10). The correlation coefficients between the total score of the Cancer Module and the Generic Core Scales were over 0.70 for both the child self-reports and the parent proxy-reports. However, correlation coefficients between the 'procedural and treatment anxiety' and 'social functioning' subscales in the child self-reports were weak. For both child reports and parent reports, 'pain and hurt' and 'nausea' subscales showed the strongest correlation with the 'physical health' subscale. For children, the 'procedural anxiety' and 'worry' subscales were strongly correlated with 'physical health' and 'emotional functioning'; the 'cognitive problems' subscale was strongly correlated with 'school functioning'; and 'perceived physical appearance' and communication' subscales were strongly correlated with the 'social functioning' subscale. For parents, all subscales except 'pain and hurt' and 'nausea' subscales showed a strong correlation with the 'emotional functioning' subscale.

**Table 10:** Spearman's Correlation Coefficients between the PedsQL Cancer Module and the PedsQL Generic Core Scales

	PedsQL Generic Core Scales				
PedsQL Cancer Module	Physical health	Emotional functioning	Social functioning	School functioning	Total
Child self-report					
Pain and hurt	0.51**	0.45**	0.30**	0.31**	0.52**
Nausea	0.57**	0.48**	0.38**	0.36**	0.54**
Procedural anxiety	0.37**	0.30**	0.36**	0.14	0.35**
Treatment anxiety	0.17*	0.17*	0.30**	0.12	0.24**
Worry	0.52**	0.53**	0.33**	0.37**	0.58**
Cognitive problems	0.49**	0.53**	0.49**	0.59**	0.63**
Perceived physical appearance	0.51**	0.58**	0.44**	0.33**	0.58**
Communication	0.43**	0.42**	0.49**	0.38**	0.54**
Total	0.67**	0.66**	0.58**	0.48**	0.76**

Parent proxy-report					
Pain and hurt	0.49**	0.44**	0.25**	0.25**	0.47**
Nausea	0.62**	0.56**	0.26**	0.33**	0.50**
Procedural anxiety	0.37**	0.45**	0.30**	0.16*	0.36**
Treatment anxiety	0.29**	0.43**	0.30**	0.20**	0.38**
Worry	0.39**	0.45**	0.21**	0.32**	0.47**
Cognitive problems	0.32**	0.43**	0.39**	0.43**	0.51**
Perceived physical appearance	0.42**	0.50**	0.28**	0.22**	0.52**
Communication	0.39**	0.47**	0.31**	0.23**	0.44**
Total	0.65**	0.71**	0.44**	0.38**	0.70**

*P = < 0.05, **P = < 0.01 (2-tailed)

Second, the correlations between the PedsQL scale scores and child self-rating depression screening scores (DSRS-C or CES-D) were examined (Table 11). For the children who were considered depressed, both the DSRS-C and CES-D scores were strongly correlated with the 'emotional functioning' score and total score of the Generic Core Scales. For children aged 8 to 15 years, DSRS-C scores were strongly correlated with 'procedural anxiety,' 'worry,' 'perceived physical appearance,' and 'communication' scores, and the total score of the Cancer Module. For children aged 16 to 18 years, CES-D scores were moderately correlated with 'treatment anxiety' and 'communication' scores of the Cancer Module. Both DSRS-C and CES-D scores of children were strongly correlated with the total score of their parent's CES-D scores (correlation coefficient: 0.986 for DSRS-C, and 0.771 for CES-D; data not shown).

**Table 11:** Spearman's Correlation of the PedsQL child self-report with DSRS-C and with CES-D

	Depression scale	
	DSRS-C score > = 16	CES-D score > = 16
PedsQL Generic Core Scales		
Physical health	-0.636	-0.290
Emotional functioning	-0.815*	-0.883*
Social functioning	-0.849**	-0.202
School functioning	-0.617	-0.138
Total	-0.704	-0.775*

PedsQL Cancer Module		
Pain and hurt	-0.208	0.200
Nausea	-0.598	-0.257
Procedural anxiety	-0.811*	0.274
Treatment anxiety	-0.185	-0.397
Worry	-0.916**	-0.373
Cognitive problems	-0.556	-0.378
Perceived physical appearance	-0.849*	-0.294
Communication	-0.729	-0.486
Total	-0.889**	-0.371

*P = < 0.05, **P = < 0.01 (2-tailed)

CES-D: Center for Epidemiologic Studies Depression scale DSRS-C: Depression Self-Rating Scale for Children

For clinical validity, we compared the total and subscale scores between on-treatment and off-treatment status by Kruskal-Wallis and Mann-Whitney U tests (Table 12) because only treatment status was a significant factor among patients' characteristics for the total score of the PedsQL Cancer Module (Table 2). Off-treatment status was divided into 2 groups ( = < 12 mo and > 12 mo) and analyzed separately.

**Table 12:** Clinical validity of the PedsQL Cancer Module: Comparison of scores by treatment status

PedsQL Subscales	Children				Parents			
	
	Mean n Rank	Difference	Kruskal Wallis Test	*P *value	Mean n Rank	Difference	Kruskal Wallis Test	*P *value
**Pain and hurt**		a,c**	10.392	0.006		a,c***, b,c*	21.296	0.000
On Tx_(a)_	63 85.63				87 97.70			
Off Tx = < 12_(b)_	27 91.06				33 113.92			
Off Tx > 12_(c)_	110 111.33				120 138.84			
**Nausea**		a,c***, b,c***	66.648	0.000		a,b*, b,c***, a,c***	88.814	0.000
On Tx_(a)_	64 61.97				82 68.57			
Off Tx = < 12_(b)_	26 74.13				32 99.81			
Off Tx > 12_(c)_	107 127.19				117 153.67			
**Procedural anxiety**		a,c**	8.225	0.016		a,c***, b,c*	12.438	0.002
On Tx_(a)_	65 86.58				85 103.49			
Off Tx = < 12_(b)_	27 94.31				33 107.65			
Off Tx > 12_(c)_	109 111.25				122 135.82			
**Treatment anxiety**			3.279	0.194		a,b*, a,c***	12.013	0.002
On Tx_(a)_	64 99.73				84 100.32			
Off Tx = < 12_(b)_	27 88.19				33 127.80			
Off Tx > 12_(c)_	110 104.88				122 131.44			
**Worry**		a,c***, b,c*	26.914	0.000		a,c***	14.792	0.001
On Tx_(a)_	63 73.54				85 100.80			
Off Tx = < 12_(b)_	27 89.44				33 112.21			
Off Tx > 12_(c)_	110 118.65				122 136.47			
**Cognitive problems**			1.367	0.505			3.323	0.190
On Tx_(a)_	63 93.13				86 110.42			
Off Tx = < 12_(b)_	27 101.78				33 131.41			
Off Tx > 12_(c)_	109 103.53				122 125.64			
**Perceived physical appearance**			1.287	0.525		a,c*	4.944	0.084
On Tx_(a)_	65 96.07				86 109.20			
Off Tx = < 12_(b)_	27 97.52				33 117.06			
Off Tx > 12_(c)_	110 105.69				122 130.38			
**Communication**		a,c*	6.392	0.041		a,c***	11.325	0.003
On Tx_(a)_	65 90.70				84 102.44			
Off Tx = < 12_(b)_	27 89.17				33 111.58			
Off Tx > 12_(c)_	110 110.91				122 134.37			

On Tx: on treatment sample; Off Tx = < 12: off treatment = < 12 months sample; Off Tx > 12: off treatment > 12 months sample.

* P < 0.05, **P = < 0.01, ***P = < 0.001 by Mann-Whitney U test.

Children who had been off treatment over 12 months and their parents demonstrated significantly higher scores than those on treatment except for 'cognitive problems' and 'perceived physical appearance' subscales. On the other hand, physical and emotional quality of life scores associated with anti-cancer treatment were significantly improved among them.

Social and school functioning subscales, such as 'cognitive problems' and 'perceived physical appearance' had not improved long after the completion of treatment, and 'communication' scores of children had not improved within 12 months of completion of treatment.

### Feasibility

The percentage of missing values was 0.68% for child self-reports and 0.98% for parent proxy reports. According to the pilot testing, the time required to complete the questionnaires was estimated to be 5 to 10 minutes (median, 8 min) for the child self-report and 2 to 5 minutes (median, 3 min) for the parent proxy report. This would be enough to demonstrate the feasibility of the Japanese version of the PedsQL 3.0 Cancer Module.

## Discussion

The present study demonstrated the reliability, validity, and feasibility of the Japanese version of the PedsQL Cancer Module. The guardians who answered the questionnaires were much older than the Brazilian subjects [16], it may reflect the rising age at first birth among Japanese women.

For internal consistency, Cronbach's coefficient alpha for the overall scale exceeded 0.70 except for the 'pain and hurt,' 'cognitive problems,' 'perceived physical appearance,' and 'communication' subscales in child self-reports for children aged 5 to 7 years. The Cronbach's coefficient alpha ranged from 0.53 to 0.67 in these subscales. The same tendency was shown in the original English version (0.38 to 0.63) [11]. The reason may be that children under the age of 7 years can only describe the general amount of pain they feel. Therefore, it is sometimes difficult to accurately measure the level of pain even using very simple scales [23]. As Dr. James W. Varni mentioned [11], child self-report scales that cannot achieve 0.70 should be used only for descriptive or exploratory analyses and further testing is needed for practical use.

For test-retest reliability, patients were selected who were considered to be stable and were not expected to change before completing the questionnaires for the second time. Patients did not receive treatment between the first and second completions of the questionnaires. The ideal length of the interval between the first and the second tests was not determined. A period of 2 to 14 days in considered adequate [24-27], so we used a 7-day interval in this study. ICC values among children were good to excellent, except for 3 subscales. First, for the 'treatment anxiety' subscale in 5- to 7-year-olds, the children gave the same answer for the second item, 'getting anxious about going to the doctor.' However, 2 other items, 'getting anxious when waiting to see the doctor' and 'getting anxious about going to the hospital' might be difficult to explain to young children. Test-retest reliability coefficients for the 'pain and hurt' subscale and 'treatment anxiety' subscale in children aged 5 to 7 years were also low in the validation study of the Chinese version [17]. The German and the Brazilian versions of the PedsQL Cancer Module did not report the analysis for separate age groups. However, the total scales for each age group had moderate to high ICC values for both children and parents (> 0.70).

Second, the 'treatment anxiety' subscale for 13- to 18-year-olds also demonstrated a low ICC value because many children who had been off treatment for more than 12 months gave a different answer on the retest. However, scores on both the first test and retest were very high (first test: mean, 94.79 [SD, 8.84], range 75-100]; retest: mean, 94.05 [SD, 10.45], range 75-100) and not significantly different. We considered that the low ICC value in this age group might be due to minor differences in answers. Third, the 'worry' subscale in 8- to 12-year-olds also had a low ICC value. It may be because all the children except 1 who completed the retest were off treatment for over 12 months, so that they might have had trouble answering responses such as 'worrying about side effects from medical treatments' and 'worrying about whether or not his/her medical treatments are working.' ICC values among the parents were almost good to excellent.

For validity, exploratory factor analysis identified 7 factors for both child self-reports and parent proxy-reports in our study, even though the original English version has an 8-factor structure [11]. For children, the first item of 'worry' (worrying about side effects from medical treatments) loaded on the 'nausea' factor. This suggests that patients' worries about side effects increase when the children actually feel nauseated. The second and third items of 'worry' (worrying about whether or not his/her medical treatments are working, worrying that the cancer will reoccur or relapse) loaded on the 'communication' factor, This suggests that patients have a difficult time communicating with medical staff when they worry about treatment efficacy and/or relapse. In parent proxy-reports, the first and the second items of 'worry' loaded on the 'nausea' factor. In clinical practice in Japan, we feel many parents who have a child with cancer believe that the most effective chemotherapy should cause the worst side effects (such as nausea, stomatitis, and bone marrow suppression), so that their worry about treatment efficacy may link to the 'nausea' factor.

Spearman's correlation coefficients between the child self-reports and parent-proxy reports showed strong correlation between the same subscales (*P *= < 0.01), especially in physical health scales. We think the reason for this is that objective evaluation of physical symptoms are generally easier than emotional symptoms.

Comparing the Spearman's correlation coefficients between the PedsQL 3.0 Cancer Module and the PedsQL 4.0 Generic Core Scales, all subscales and the total score of the Cancer Module were significantly correlated with all the subscales and total score of the generic core scales for both children and their parents except between 'procedural anxiety,' 'treatment anxiety,' and 'school functioning.' Specifically, the 'physical health' subscale of the generic core scale demonstrated a strong correlation with physical, emotional, and social subscales of the Cancer Module. The scores of 'emotional functioning' were good if the children did not have much pain, nausea, or worry and did not have cognitive problems at school. A good self-image about their physical appearance correlated with good emotional and social functioning. Naturally, the 'cognitive problems' subscale of the Cancer Module showed a strong correlation with the 'school functioning' subscale of the generic core scale. For parents, a similar tendency was shown. These results suggests that physical, psychological, and social factors are related to each other. We therefore need to take a multidisciplinary approach to alleviating these types of pain in children with cancer [23].

To assess concurrent validity, we also examined the correlations between the PedsQL child self-report scores and child self-rating depression scale scores (DSRS-C: 8-15 y; CES-D: 16-18 y) among children who were considered to be depressed. It is reasonable that both the DSRS-C and CES-D scores were strongly correlated with the 'emotional functioning' score of the Generic Core Scales because direct emotional expressions were used in this subscale, such as 'I feel afraid or scared,' 'I feel sad or blue,' and 'I feel angry.' These strong correlations were compatible with the results of a previous validation study to develop a Japanese version of the PedsQL generic core scales even though the participants were healthy children [14].

For the PedsQL Cancer Module, DSRS-C scores were strongly correlated with emotional domains and the total score, but not with CES-D scores. In 2010, Kamibeppu et al [28] reported that no significant differences in depression and anxiety were seen between healthy children and childhood cancer survivors who were over 16 years old. They evaluated the children's mental status with the Japanese version of the K10 [29] (10-item self-report screening instrument for mood and anxiety disorders based on the Diagnostic and Statistical Manual of Mental Disorders-Fourth Edition [DSM-IV]) [30]. They also demonstrated that childhood cancer survivors had remarkably greater posttraumatic growth compared to healthy children and concluded that the cancer experience itself does not cause depression even though they had significantly more posttraumatic stress syndrome. This would be a probable explanation for why CES-D scores of children who were considered depressed did not correlate with any subscale of the PedsQL Cancer Module. Other factors were suspicious for depression.

Kruskal-Wallis and Mann-Whitney U tests demonstrated that physical and emotional quality of life scores associated with anti-cancer treatment were significantly improved among children who had been off treatment over 12 months. However, social and school functioning, such as 'cognitive problems' and 'perceived physical appearance' did not improve. Moreover, 'communication' scores took more than 12 months to improve. We should remember that childhood cancer survivors need continuous social support.

The percentage of missing values was 0.68% for child self-reports and 0.98% for parent proxy-reports in our study. This is similar to the original English version (0.50% for child self-reports and 1.00% for parent proxy-reports) [11]. The time required to complete the questionnaires was 5 to 10 minutes (median, 8 min) for the child self-reports and 2 to 5 minutes (median, 3 min) for the parent proxy-reports.

Although 'Treatment anxiety' subscale that showed high negative skewness and ceiling effect could be improved in the future, our Japanese version of the PedsQL Cancer Module would be feasible to use in clinical practice.

## Conclusions

This study confirmed the reliability, validity, and feasibility of the Japanese version of the PedsQL 3.0 Cancer Module. This is expected to help improve the quality of life of Japanese children with cancer because until now there has been no instrument to measure pediatric cancer-specific HRQOL. The results are comparable to those of the original version and translated versions in other countries. Therefore, this module can be used for international cooperative research to measure HRQOL in pediatric cancer patients.

## Competing interests

The authors declare that they have no competing interests.

## Authors' contributions

NT, NK, KK,** and YI conceptualized the rationale and design of the study. KK advised NT about data management for SPSS. NT, YT, WO, YY, TK, KA, KT, HN, TI, MM, JO, TK**, AM, and YI coordinated participants and settings in each hospital. After approval of each Institutional Review Board, they administered questionnaires to children with cancer and their parents and collected data. NT and EM conducted statistical analyses and drafted the manuscript. All authors read and approved the final manuscript.
